# Perception of Online Lectures among Students of a Medical College in Kathmandu: A Descriptive Cross-sectional Study

**DOI:** 10.31729/jnma.6276

**Published:** 2021-03-31

**Authors:** Bhagabat Bhattarai, Sujaya Gupta, Sirjana Dahal, Aarzu Thapa, Pooja Bhandari

**Affiliations:** 1 Department of Periodontics and Oral Implantology, Kathmandu Medical College, Bhaktapur, Nepal; 2 Department of Community and Public Health Dentistry, Kathmandu Medical College, Bhaktapur, Nepal

**Keywords:** *curriculum*, *medical education*, *online education*

## Abstract

**Introduction:**

The COVID-19 global pandemic has affected all aspects of human life, with education no exception. Online lectures have been practiced in different academic institutions around the world. The objective was to know the perception towards online lectures by the undergraduate students of a medical college.

**Methods:**

This descriptive cross-sectional study was conducted among the undergraduate students of dentistry, medicine, and nursing at Kathmandu Medical College via self-administered online questionnaire. Data were collected from November to December 2020 after ethical clearance from institutional review committee (Ref. no. 0311202002). Students who had not attended even one hour of online learning per week were excluded. Responses were collected using Google Forms which were analysed in Microsoft Excel. Descriptive statistics are presented as means, standard deviations, frequencies, and percentages.

**Results:**

Out of 318 valid questionnaires, 143 (44.97%) students agreed that online lectures were effective but 138 (43.4%) disagreed that online lectures were more useful than traditional lectures. One hundred and forty five (45.60%) found online classes difficult to concentrate and 175 (55.03%) agreed that they preferred a combination of traditional teaching and online tutorials. Only two (0.63%) students strongly agreed on excellent internet during classes and 104 (32.70%) agreed it caused economic burden. Mean age of participants was 20.75±1.538 years; 202 (63.52%) were females; online learning per week was 18.75±9.157 hours; and duration of online learning was 20.28±9.997 weeks.

**Conclusions:**

Most of the students had a positive attitude towards e-learning when compared to similar studies. Further multicentric studies with larger sample size would better demonstrate whether online education partly or fully can be effective adjunct to traditional face to face interaction.

## INTRODUCTION

The coronavirus disease (COVID-19) pandemic affected not only health but also education, business, and daily activities. Medical education and training are not useful unless the students acquire the minimum knowledge (cognitive aspect), skills (psychological/ motoric aspect), and obligatory conduct values (affective aspect).^1^

Given the needs of the academic environment where learning can progress in optimal manner, the curriculum has to be frequently revised to improve the quality of health education.^1^ The impact of the pandemic affected education at all levels and need for intelligent technology should be evaluated for future.^2,3^ Studies have shown that students perceived online classes as a supplement to live lectures and did not prefer e-teaching over face-to-face teaching during the lockdown situation.^4^ Education administration and faculty members should take necessary measures to improve e-learning for better learning during lockdown.^5,6^

The objective was to know about the perception towards online lectures by undergraduate students of medical college.

## METHODS

This descriptive cross-sectional study was conducted in the Department of Periodontics and Oral Implantology, Kathmandu Medical College (KMC), Duwakot, Bhaktapur, Nepal. The data was collected from November to December 2020 after obtaining ethical clearance from the institutional review committee of KMC (Ref. 0311202002). An informed consent was obtained from the participants before they were allowed in the questionnaire section. The undergraduate (UG) students of first year to final year who had taken online classes anytime during the past six months were taken as sample units. All the UG students of Bachelor of Science in Nursing (B.Sc. Nursing), Bachelor in Nursing (BN), Bachelor of Dental Surgery (BDS), and Bachelor of Medicine, Bachelor of Surgery (MBBS) academic programs were invited to participate in the study. Students during internship were not taken into consideration as study subjects. The students who had not attended at least one hour per week of online learning were excluded from the study. Convenience sampling method was done to reach sample size. A sample size of 317 was calculated using following formula:

$$\begin{array}{ll} \text{n} & ={\text{Z}}^{\text{2}}\times \text{p}\times \text{q}/{\text{e}}^{\text{2}}\\ & ={\left(1.96\right)}^{2}\times (0.554)\times (1-0.554)/{\left(0.05\right)}^{\text{2}}\\ & =384.16 \end{array}$$

For finite population,

$$\begin{array}{ll} \text{n} & ={\text{n}}_{\circ }{\text{/[1+\{(n}}_{\circ }-1\text{),N\}]}\\ & =384.16/[1+\{384.16-1)/864]\\ & =266.136 \end{array}$$

Where,

n = required sample size
Z = 1.96 at 95% Confidence Interval (CI)
p = past prevalence, 55.4%^6^
q = 1-p
e = margin of error, 5%
N = Total undergraduate medical students, 864

After adding 20% for non-response, sample size of 319 was calculated and we collected data from 318 participants. Data were collected electronically via Google Forms. Links to the forms were shared with all 864 students from the sampling frame via Viber. The questionnaire consisted of two parts: i) informed consent and ii) proforma with demographic details and questions. Informed consent was mandatory before proceeding to the questionnaire section. Only after they had read and clicked on the informed consent page, were they allowed access to the proforma section.

Data from Google forms were opened with Microsoft Excel Sheet and analysed. Descriptive statistics have been presented as means, standard deviations, frequencies, and percentages.

## RESULTS

Out of total 318 valid questionnaires analysed, 151 (47.49%) participating students agreed that online lectures were helpful to their learning (Table 1). About the moderators/application systems like Avyaas, Zoom, most 180 (56.6%) felt that applications were helpful in communication and interaction with teachers and other students. Almost half 168 (52.83%) students accepted and only 7 (2.20%) strongly disagreed about feeling comfortable exploring online tutorials. A total of 269 (84.59%) responded that online tutorials needed further improvement to support their learning. Similarly, 138 (43.40%) students disagreed that online lectures were more useful than traditional lectures. Nearly half 225 (70.76%) students found online classes difficult to concentrate on and 208 (65.4%) disagreed that online tutorials should replace traditional lectures and live demonstrations. More than half 245 (77.04%) preferred a combination of traditional teaching and online tutorials. Only two (0.63%) students strongly agreed that the quality of internet connection was excellent for online lectures and many 156 (49.05%) students agreed that internet classes caused economic burden.

**Table 1:** Responses of the student participants on the Likert scale.

Questions	Strongly agree n (%)	Agree n (%)	Uncertain n (%)	Disagree n (%)	Strongly disagree n (%)
1. Online lectures were helpful to my learning	8 (2.52)	143 (44.97)	95 (29.87)	57 (17.92)	15 (4.72)
2. Moderators/Application systems like Avyaas, Zoom, etc. were helpful in communication and interaction with teachers and other students	10 (3.14)	170 (53.46)	76 (23.90)	49 (15.41)	13 (4.09)
3. I feel comfortable exploring online tutorials	14 (4.40)	154 (48.43)	81 (25.47)	62 (19.50)	7 (2.20)
4. Online tutorials need further improvement to support my learning	83 (26.10)	186 (58.49)	36 (11.32)	6 (1.89)	7 (2.20)
5. Online lectures were more useful than traditional lectures	4 (1.26)	50 (15.72)	59 (18.55)	138 (43.40)	67 (21.07)
6. I found online classes difficult to concentrate on	80 (25.16)	145 (45.60)	40 (12.58)	43 (13.52)	10 (3.14)
7. Online tutorials should replace traditional lectures and live demonstrations	15 (4.72)	46 (14.47)	49 (15.41)	131 (41.19)	77 (24.21)
8. I prefer a combination of traditional teaching and online tutorials	70 (22.01)	175 (55.03)	38 (11.95)	22 (6.92)	13 (4.09)
9. Quality of internet connection was excellent	2 (0.63)	44 (13.84)	68 (21.38)	124 (38.99)	80 (25.16)
10. Internet classes caused economic burden	52 (16.35)	104 (32.70)	74 (23.27)	71 (22.33)	17 (5.35)

**Figure 1.**
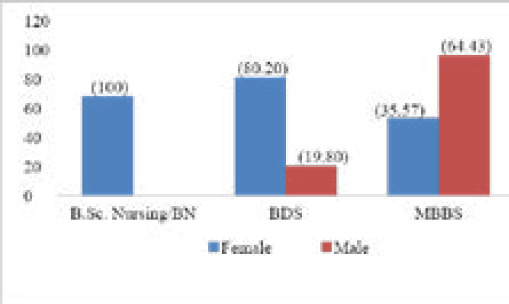
Sex distribution of the participants in different academic programs.

The mean age of the participants was 20.75±1.538 years (standard error of mean, SEM=0.086; median=20; mode=20) with minimum of 18 years and maximum of 26 years. Among the participants there were 202 (63.52%) females and 116 (36.48%) males. Most participants were female as all of the nursing students and majority of the BDS students were female (Figure 1).

Almost half 149 (46.86%) students were of MBBS academic program followed by BDS, and B.Sc. Nursing/BN programs (Table 2).

**Table 2:** Distribution of students according to academic year and program.

Academic Year	B.Sc. Nursing / BN n (%)	BDS n (%)	MBBS n (%)	Total n (%)
First	17 (5.35)	12 (3.77)	88 (27.67)	117 (36.79)
Second	15 (4.72)	45 (14.15)	54 (16.98)	114 (35.85)
Third	26 (8.18)	8 (2.52)	6 (1.89)	40 (12.58)
Fourth	10 (3.14)	17 (5.35)	1 (0.31)	28 (8.81)
Fifth	-	19 (5.97)	-	19 (5.97)
**Total**	**68 (21.38)**	**101 (31.76)**	**149 (46.86)**	**318 (100)**

The online learning hours per week were 18.75±9.157 hours (SEM=0.513; median=18; mode=18) with minimum of one hour and maximum of 48 hours per week. The duration of online learning of the participants was 20.28±9.997 weeks (SEM=0.561; median=20; mode=18) with the learning going on for a minimum of one week to a maximum of 36 weeks. Almost all 306 (96.23%) participants used Zoom for online learning (Table 3).

**Table 3:** Medium used for online education.

Application used for online lecture	Frequency n (%)
Avyaas	4 (1.26)
Google Meet	4 (1.26)
Microsoft Teams	3 (0.94)
Viber	1 (0.31)
Zoom	306 (96.23)
**Total**	**318 (100)**

## DISCUSSION

Currently, the world is responding to a pandemic of contagious respiratory disease caused by a novel coronavirus, named COVID-19. In this circumstance, online lecture was better option to avoid gathering. Dental educators now have the capabilities and technologies to modernise their approaches.^7^ Textbooks can be turned into e-textbooks which is helpful in acquisition of knowledge. Videos can be helpful for improving psychomotor skills. 'Synchronous'' and asynchronous communication through the internet is helpful in developing attitude. Assessments, for example multiple choice questions (MCQ) can be done online. Hence, online education is another useful instrument in the teaching toolbox.^8^ Digitalisation offers great potential to revolutionise dental education to help prepare future dentists for their daily practice.^9^ There are conflicting results on improvement in students' performance.^10^ COVID-19 pandemic trajectory has disrupted routines in hospitals, medical schools, and beyond. There was a pause in clinical posting, conference presentation to examination. The panic situation is felt by students as well as faculties.

Chavarría-Bolaños et al. suggest it was necessary to categorise the academic courses depending on their virtualisation's possibility (curricula analysis and classification), to better understand the extent of the impact and the work needed to contain, as far as the possibilities are allowed. Teachers needed further training in the application of virtual strategies which they had not used before.^11^

In the current study, majority of the respondents were from first and second year. This may be due to clinical posting and less lectures in third, fourth, and final years compared to first and second years. The study survey also solicited participants' feedback regarding their experience in online education. Of all, 44.97% of the respondents agreed that online education was helpful whereas 29.87% were uncertain. A study done by Rajab et al. showed that 76% of participants intended to integrate the online expertise garnered during the pandemic into their practice.^12^

A similar study on students of Master of Public Health (MPH) and MBBS demonstrated a largely positive impact on online medical education during COVID-19 pandemic.^12^ Dental students of Pakistan unanimously voiced dissatisfaction toward various elements of online teaching sessions.^13^ Satisfaction with this method of education within the students is good, but not yet suitable for most of medical disciplines at biomedical faculties in Bosnia and Herzegovina.^1^ Online courses might not be reliable learning methods.^14^ In this study 138 (43.40%) disagreed on the usefulness of online lectures compared to traditional lectures. A similar study done by Gupta et al. showed 55.4% students disagreed that online classes were more effective.^6^ A study by Abbasi et al. showed that 63% students disagreed on usefulness of online education.^5^ It may be due to inefficient internet quality. In current study, only 2 (0.63%) agreed on excellent connections.

Nearly half 45.60% of the respondents reported difficulty in concentration during the online classes.

The respondents also suggested improving online tutorials 186 (58.49%). This may be due to poor knowledge of information technology to the trainer or poor internet connection. A study by Abbasi et al. showed 77% students had negative perceptions towards e-learning.^5^

Instructors and faculty members must possess and master all technical achievements and new advancements offered by e-learning.^15^ More specific study has shown that type of education had a significant effect on the theoretical test score (P <0.001) but had no significant effect on the clinical score (P=0.072).^16^ Other studies have showed 48.6%^17^ and 76%^12^ of the participants intended to integrate the traditional lecture mixed with online learning whereas in this study, 70 (22.01%) of students strongly agreed and 55.03% of students agreed on combination of both traditional and online education. As suggested by Choules, we can go forward for change in current syllabus after through re-evaluation and further study.^8^

Limitation of this study was that the perceptions of quality of online tutors were not measured and this study was conducted at a single institution with limited sample size.

## CONCLUSIONS

Student participants of this study had a positive attitude for online learning though they lacked concentration. In order to increase the effectiveness of online education, the online tutorials as well as internet connection may be improved. Further multicentric studies with larger sample size would better demonstrate whether online education partly or fully can be effective adjunct to traditional face to face interaction.

## References

[1] Masic I (2008). E-learning as new method of medical education.. Acta Inform Med..

[2] Chang TY, Hong G, Paganelli C, Phantumvanit P, Chang WJ, Shieh YS (2021). Innovation of dental education during COVID-19 pandemic.. J Dent Sci..

[3] Deery C (2020). The COVID-19 pandemic: implications for dental education.. Evid Based Dent..

[4] Asiry MA (2017). Dental students' perceptions of an online learning.. Saudi Dent J..

[5] Abbasi S, Ayoob T, Malik A, Memon SI (2020). Perceptions of students regarding E-learning during Covid-19 at a private medical college.. Pak J Med Sci.

[6] Gupta A, Shrestha RM, Shrestha S, Acharya A, Pandey N (2020). Perception of BDS students of Kathmandu University on online learning during COVID-19 pandemic.. Orthod J Nepal..

[7] Alzahrani SB, Alrusayes AA, Aldossary MS (2020). Impact of COVID-19 pandemic on dental education, research, and students.. Int J Health Sci Res..

[8] Choules AP (2007). The use of elearning in medical education: a review of the current situation.. Postgrad Med J..

[9] Zitzmann NU, Matthisson L, Ohla H, Joda T (2020). Digital undergraduate education in dentistry: A systematic review.. Int J Environ Res Public Health..

[10] Khasawneh R, Simonsen K, Snowden J, Higgins J, Beck G (2016). The effectiveness of e-learning in pediatric medical student education.. Med Educ Online..

[11] Chavarría-Bolaños D, Gómez-Fernóndez A, Dittel-Jiménez C, Montero-Aguilar M (2020). E-Learning in dental schools in the times of COVID-19: A review and analysis of an educational resource in times of the COVID-19 pandemic.. Odovtos-Int J Dent Sci..

[12] Rajab MH, Gazal AM, Alkattan K (2020). Challenges to online medical education during the COVID-19 pandemic.. Cureus.

[13] Sarwar H, Akhtar H, Naeem MM, Khan JA, Waraich K, Shabbir S (2020). Self-reported effectiveness of e-learning classes during COVID-19 pandemic: A nation-wide survey of Pakistani undergraduate dentistry students. Eur J Dent.

[14] Aboalshamat KT, Banjar AM, Al-Jaber MI, Turkistani NM, Al-Amoudi MT (2019). The effectiveness of online course intervention to improve knowledge of antimicrobial resistance among dental students, in comparison to reference group using a randomized controlled trial.. Open Access Maced J Med Sci..

[15] El-Seoud MSA, Taj-Eddin IATF, Seddiek N, El-Khouly MM, Nosseir A (2014). E-learning and students' motivation: A research study on the effect of e-learning on higher education.. International Journal Emerging Technologies in Learning (iJET)..

[16] Soltanimehr E, Bahrampour E, Imani MM, Rahimi F, Almasi B, Moattari M (2019). Effect of virtual versus traditional education on theoretical knowledge and reporting skills of dental students in radiographic interpretation of bony lesions of the jaw.. BMC Med Educ..

[17] Turkyilmaz I, Hariri NH, Jahangiri L (2019). Student's perception of the impact of e-learning on dental education.. J Contemp Dent Pract..

